# Microtubule asters as templates for nanomaterials assembly

**DOI:** 10.1186/1754-1611-6-23

**Published:** 2012-12-27

**Authors:** Vivek Verma, Jeffrey M Catchmark, Nicole R Brown, William O Hancock

**Affiliations:** 1 Department of Engineering Science and Mechanics, Pennsylvania State University, University Park, PA 16802, USA; 2 Present Address: Department of Materials Science and Engineering, Indian Institute of Technology Kanpur, Kanpur, India; 3 Agricultural and Biological Engineering, Pennsylvania State University, University Park, PA 16802, USA; 4 School of Forest Resources, Pennsylvania State University, University Park, PA 16802, USA; 5 Bioengineering Department, Pennsylvania State University, University Park, PA 16802, USA

## Abstract

Self organization of the kinesin-microtubule system was implemented as a novel template to create percolated nanofiber networks. Asters of microtubule seeds were immobilized on glass surfaces and their growth was recorded over time. The individual aster islands became interconnected as microtubules grew and overlapped, resulting in a highly percolated network. Cellulose nanowhiskers were used to demonstrate the application of this system to nanomaterials organization. The size distribution of the cellulose nanowhiskers was comparable to that of microtubules. To link cellulose nanowhiskers to microtubules, the nanowhiskers were functionalized by biotin using cellulose binding domains. Fluorescence studies confirmed biotinylation of cellulose nanowhiskers and binding of cellulose nanowhiskers to biotinylated microtubules.

## Background

Controlled organization of multi-component materials at the nano and micro scales offers the potential for dramatic improvements in physical, chemical, electrical and optical properties. Self organized structures are ubiquitous in nature and offer great possibilities for their use in science and engineering applications. Self organization requires energy input from the system and should not be confused with self assembly, which does not require energy from the system. One of the most important aspects of self organization has been manipulation of materials at micro and/or nano scales to create architectures that would have been difficult to synthesize using conventional fabrication techniques. Creation of mechanically percolated networks of nanoparticles or nanofibers using self organization is envisioned to uniquely enable further study of such systems.

Favier et al. observed nearly three orders of magnitude increase in shear modulus of latex following incorporation of 6 wt% cellulose nanowhiskers [1]. The model suggested that this behavior is governed by a mechanical percolation mechanism which occurs at a certain threshold of fiber content. However, the percolation can also be achieved by controlling the organization of cellulose nanowhiskers at the microscale. The kinesin – microtubule system can be used to study the effects of such organization in this and many other systems.

Biomolecular machinery, consisting of microtubules and its associated proteins (kinesin and dynein) participate in intracellular transport and play an important role in cell division by forming a mitotic spindle [2]. Microtubules are protein filaments that are 25 nm in diameter and can be tens of micrometers long. The motor proteins kinesin and dynein ‘walk’ along microtubules constituting a two way transport system. During cell division, microtubules and motor proteins organize into a bipolar spindle and work together to segregate chromosomes to the emerging daughter cells [3].

The kinesin – microtubule system has been shown, *in vitro,* to form self organizing structures [4-6] and has also been used to carry silicon microchips [7]. For example, this ‘nano machinery’ has been used by Nédélec et al (1997) to organize microtubules into asters *in vitro*[4]. Others have developed tools for immobilizing microtubules in novel geometries *in vitro* for transport applications and for studying mitotic spindle function [7-10]. To date, however, no work has been done to implement these systems as templates for nanomaterials organization.

In this work, the use of self-organized microtubule architectures as a template to organize nanomaterials was demonstrated using cellulose nanowhiskers. Commercial cellulose (CF 11, Whatman) was hydrolyzed to create nanowhiskers and these were bound to microtubules using biotin-avidin and cellulose binding domain proteins [11-13].

## Methods

### Cellulose hydrolysis

Fibrous, medium cellulose powder (CF 11, Whatman) was hydrolyzed to obtain cellulose nanowhiskers. CF 11, prior to hydrolysis, was composed of fibers ~125-350 μm in length and less than 10 μm in diameter. The parameters for hydrolysis were taken primarily from Bondeson et al [14]. In summary, 100 gm cellulose was added to 0.1 M sodium hydroxide (NaOH) and stirred for 2 hours to remove any surfactants. Sodium hydroxide was then removed using five centrifuge-wash cycles with deionized water. For cellulose hydrolysis, 63.5% w/w sulfuric acid (Mallinckrodt Inc) solution in water was prepared in deionized (DI) water and the temperature was maintained at 35°C on hot plate with stirrer. Cellulose was added and stirred for 90 minutes while maintaining the temperature between 42-47°C. The reaction was stopped by placing the flask on ice and letting the temperature drop to 15°C. The solution was then centrifuged (10 min, 3026 × g, 5°C) and washed with deionized water to remove excess acid and was subsequently sonicated to separate nanowhiskers into individual fibers. This cycle was repeated 5 times to raise the pH above 5. Potassium hydroxide (KOH) was then used to adjust the pH to neutral. Resulting cellulose nanowhiskers were analyzed by field emission scanning electron microscope (FESEM), LEO 1530, for size distribution. For FESEM characterization, 100 μl of cellulose nanowhisker suspension was dispensed onto a gold coated microscope slide. The slide was then baked at 37°C overnight to remove moisture. Finally, gold was sputter coated on the dried cellulose nanowhiskers to prevent charging. Similarly, hydrolysis was carried out for 60 and 120 minutes to study the effect of time on the length distribution of hydrolyzed cellulose nanowhiskers. The cellulose nanowhiskers obtained were biotinylated by incubating them in the presence of cellulose binding domain proteins, followed by centrifugation and resuspension to remove any unbound protein.

### Cellulose biotinylation

Acid hydrolyzed and pH-balanced cellulose nanowhiskers were centrifuged and resuspended in BRB80 buffer (80 mM PIPES (piperazine-N,N’-bis(2-ethanesulfonic acid), 1 mM MgCl_2_, 1 mM EGTA (ethylene glycol tetra-acetic acid), pH 6.8) before biotinylation. Cellulose nanowhiskers were biotinylated by mixing 200 ml of suspended cellulose nanowhiskers (5.2 mg/ml) with 12 mg biotin (sulfosuccinimidyl-6-[biotinamido]-6-hexanamidohexanoate, Pierce) and 1.7 mg cellulose binding domain (Sigma). Cellulose binding domains bind strongly to cellulose and covalently bind to biotin; hence, they were used as a linker to bind cellulose and biotin. The solution was mixed for 4 hours on a laboratory rotor and then dialyzed for 2 hours in BRB80 buffer to remove unbound biotin. The cellulose solution was again dialyzed in BRB80 buffer at 4°C for overnight. We refer to the resulting samples as biotinylated cellulose nanowhiskers. Fluorescence microscopy studies were performed using a Nikon E600, 100×, 1.3 N. A. Plan Fluor objective on the biotinylated cellulose to confirm biotinylation. Biotinylated cellulose nanowhiskers obtained were aliquoted and stored at -20°C. For fluorescence microscopy experiments, biotinylated cellulose nanowhiskers were mixed with alexafluor 647 labeled streptavidin, and any unbound streptavidin was removed by washing with BRB80 buffer.

### Aster formation from MT seeds

**Figure 1:**
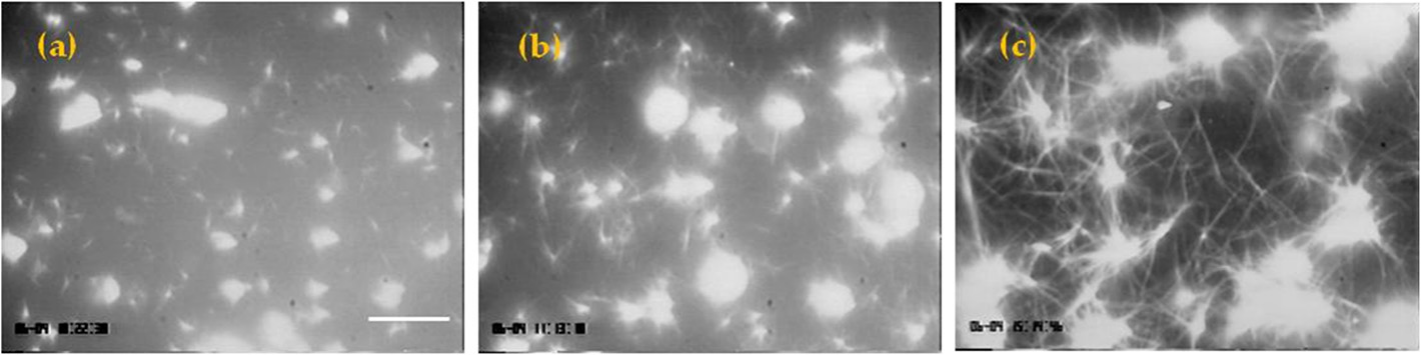
**Polymerization of asters to form microtubule templates with varying degrees of percolation.** Polymerization was conducted for (**a**) 10 minutes, (**b**) 40 minutes, and (**c**) 130 minutes. As microtubules grew they became interconnected and after 130 minutes a fully percolated system emerged. The frame size of (**a**), (**b**) and (**c**) is the same. The scale bar is 10 μm.

Microtubule seeds were polymerized using rhodamine labeled tubulin. Tubulin was purified from bovine brain and was labeled with rhodamine using standard procedures [15,16]. To polymerize microtubules, 32 μM rhodamine tubulin, 1 mM GTP (guanosine triphosphate), 4 mM MgCl_2_ and 5% DMSO (dimethyl sulfoxide) were combined in BRB80 buffer, and the temperature was raised to 37°C for 20 minutes. Polymerized microtubules were then diluted 100-fold in BRB80 and 10 μM paclitaxel to stabilize them, resulting in a population of microtubules with lengths in the range of 5-20 μm. Microtubule seeds were prepared by shearing microtubules using 30-gauge needle and followed by centrifuging them to remove free tubulin. Microtubule seeds were then mixed with biotinylated kinesin (50 μg/ml), 3 μg neutravidin (Invitrogen), 40 μg casein (Sigma Inc.), 0.7 mM ATP and left for 10 minutes to produce asters of microtubule seeds. BRB80 with 20 mM D-glucose, 20 μg/ ml glucose oxidase, 8 μg/ml catalase and 0.5% β-merceptoethanol was used as antifade reagent. Microtubule seeds were flowed into a flow chamber and free tubulin was introduced to extend the microtubule seeds. The flow cells containing microtubule seeds and free tubulin below the critical concentration for microtubule nucleation were incubated at 37°C to allow microtubule extension. The growth of microtubules over time (Figure 1) was visualized by epifluorescence microscopy (Nikon E600, 100×, 1.3 N. A. Plan Fluor objective, TRITC filter cube with emission centered at 630 nm).

### Flow cells to visualize individual microtubules binding to cellulose

Flow cells were made using APTES-coated glass cover slips and kinesin was incubated in the flow cells in presence of casein. Biotinylated microtubules and casein were then introduced in the presence of adenylyl imidodiphosphate (AMP-PNP), a non-hydrolysable analogue of ATP. Unbound microtubules were washed with 0.5 mg/ml bovine serum albumin (BSA) followed by injecting unlabeled (without any fluorophore) neutravidin. BSA was used to prevent non-specific binding of neutravidin to the glass surface. Biotinylated cellulose nanowhiskers solution was then injected into the flow cell followed by alexa-flour 647 conjugated streptavidin. Flow cells were washed with antifade solution to remove any unbound streptavidin and to prevent bleaching of the dye during observation.

### Flow cells to confirm binding of biotinylated microtubules to biotinylated cellulose nanowhiskers

Biotinylated cellulose nanowhiskers were introduced into a flow cell constructed with an APTES-coated glass cover slip, followed by flowing in a solution containing 2 mg/ml BSA and 0.2 mg/ml casein to block nonspecific binding of neutravidin to the surface. Next, unlabeled neutravidin was injected into the flow cell and allowed to bind to the biotinylated cellulose. The flow cell was then washed with BRB80 buffer followed by a wash with the BSA and casein solution.

## Results and discussion

Microtubule asters can be created via a self organizing mechanism where organization at the micro-scale is driven by multi-headed biomotor constructs powered by adenosine triphosphate (ATP) hydrolysis [4]. Percolated microtubule networks can be formed using these asters by further polymerization of the microtubules after aster formation. This process is shown in Figure 1 where polymerization of microtubules for 130 minutes resulted in interconnected asters and a percolated network. The key in using such systems for studying the impact of nanoscale assembly is linking a given nanomaterial to the microtubule template. This process is demonstrated here using cellulose nanowhiskers, although it can be applied to many other systems.

Biotinylation of protein is a standard procedure widely used in the biotechnology field. The binding constant between biotin and avidin is among the highest measured in biological systems, enabling the effective binding of two ligands labeled with these components. Beyond proteins, other materials, even non-biologically derived materials, can be connected using this system through intermediate proteins. Antibodies have been generated for a wide array of biological and synthetic materials, which in turn can be labeled with biotin. In some cases, specific binding proteins are already known that can be produced in needed quantities through genetic transformation of microbial system (such as the bacterium *E. coli*) to express the protein, microbial fermentation and protein purification. Cellulose binding domain proteins, which are an integral component of cellulose degrading enzymes, have been produced using such procedures. Cellulose binding proteins exist that even target specific regions of the cellulose surface including the highly ordered surfaces and reducing or non-reducing ends. The availability of cellulose binding proteins makes cellulose nanowhiskers an ideal model system for demonstrating the use of microtubule templates for organizing nanomaterials.

**Figure 2:**
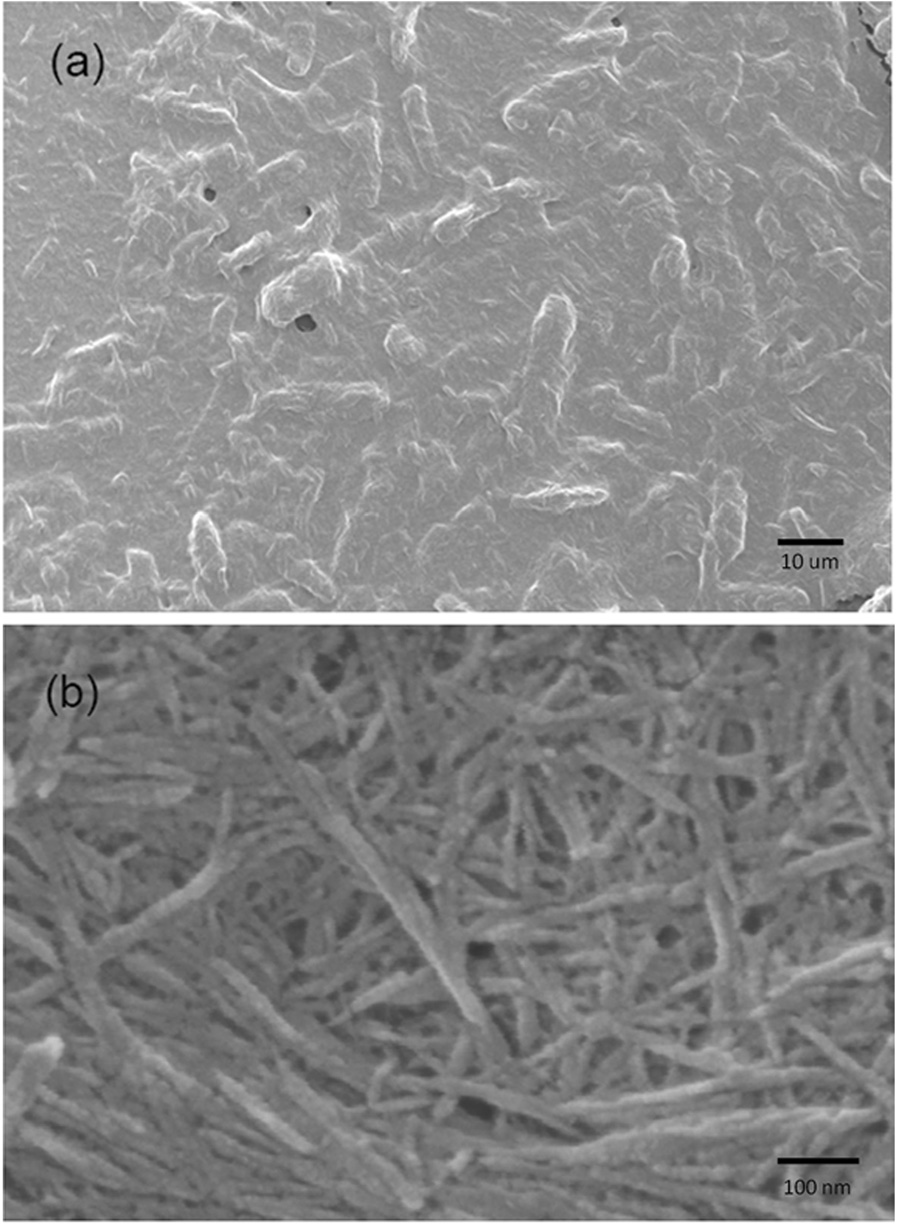
**FESEM images of cellulose hydrolyzed by sulfuric acid for 90 min (a) bottom of suspension (b) top of suspension.** Cellulose suspension was dried overnight in oven at 37°C to remove moisture. The resulting film was sputter coated with gold to prevent charging. FESEM accelerating voltage was 2 kV. Scale bar for (**a**) is 10 μm and for (**b**) is 100 nm.

To generate cellulose nanowhiskers, commercially available cotton cellulose (CF 11, Whatman) was acid hydrolyzed and suspended in deionized water after acid removal and pH balance steps. When left undisturbed for 2-3 days, the cellulose nanowhiskers solution separated into top and bottom regions, where the nanowhiskers located in the bottom fraction were larger and settled by gravity. To assess the dimensions of these fractions, samples from the top and bottom regions of the suspension were analyzed using a field emission scanning electron microscope (FESEM). The bottom part of the suspension contained larger particles of cellulose (at least 20 μm long) (Figure 2a), while the upper suspension contained cellulose nanowhiskers that were less than 5 μm long and less than 50 nm in diameter (Figure 2b). Longer hydrolysis time resulted in a higher percentage of cellulose nanowhiskers. Hydrolysis time was optimized to 90 minutes, which provided the size distribution of the cellulose nanowhiskers on the same order as that of microtubules.

**Figure 3:**
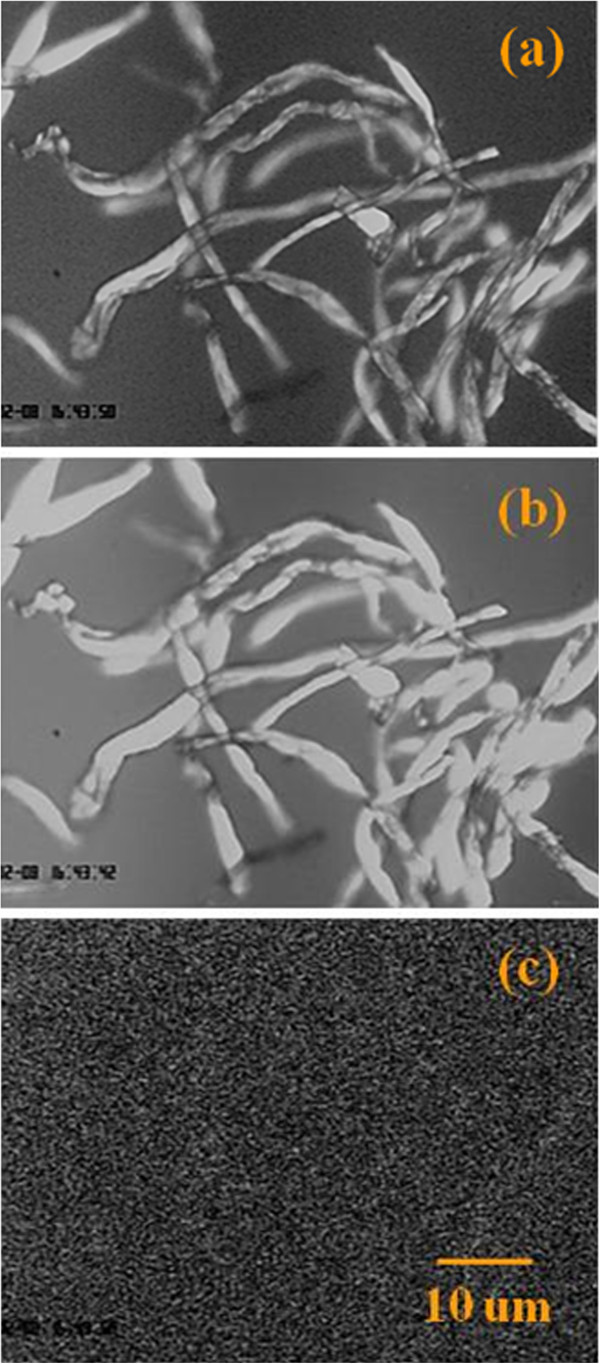
**Characterization of biotinylated microcrystalline cellulose fibers.** Biotinylated cellulose fibers were treated with alexafluor 647 streptavidin to verify the biotinylation process. Differential interference contrast (DIC) and fluorescence microscopy were used. (**a**) Microcrystalline cellulose observed under DIC. (**b**) Fluorescence was observed when microcrystalline cellulose fibers were observed under a Cy5 filter (emission centered at 699 nm). (**c**) To rule out auto fluorescence of cellulose or background fluorescence, the streptavidin conjugated fibers were observed under a FITC filter (emission centered at 535 nm). No fluorescence was observed under FITC filter (as well as TRITC/Rhodamine/CY3 and DAPI/Hoechst filters, data not shown) indicating absence of auto fluorescence or background fluorescence. The frame size of (**a**), (**b**) and (**c**) is the same. The scale bar is 10 μm for all images.

Fluorescence microscopy was then used to study the effectiveness of cellulose biotinylation. Figure 3a depicts microcrystalline cellulose observed under simple differential interference contrast (DIC) microcopy. The cellulose was then treated with alexafluor 647 streptavidin, which would bind strongly to the biotinylated cellulose. Figure 3b shows the fluorescence emission observed through a Cy5 filter indicating a strong fluorescence signal associated with the alexafluor 647. To rule out auto fluorescence, the cellulose was also observed through three filters: a CFI Epi-FL Filter Block N B-2E/C (FITC/GFP) consisting of excitation filter Ex465-495, dichroic mirror DM505, and barrier filter BA515-555; a CFI Epi-FL Filter Block N G-2E/C (TRITC/Rhodamine/CY3) consisting of excitation filter Ex540/25, dichroic mirror DM565, and barrier filter BA605/55; and a CFI Epi-FL Filter Block N UV-2E/C (DAPI/Hoechst) consisting of excitation filter Ex330-380, dichroic mirror DM400, and barrier filter BA435-485. No fluorescence was observed in any of these cases. Figure 3c depicts the image obtained using the FTIC/GFP filter. As a second control un-biotinylated cellulose was mixed with alexafluor 647-tagged streptavidin, washed with BRB80 buffer, and the sample was analyzed for any fluorescence from the cellulose nanowhiskers (result not shown). There was no fluorescence observed in this case, indicating that the fluorescence signal in the biotinylated cellulose nanowhiskers was not from a background or any non-specific binding of streptavidin to the cellulose nanowhiskers. Hence, fluorescence studies showed that the cellulose nanowhiskers have been biotinylated and that they can be linked to biotinylated proteins via biotin-streptavidin linkages.

After establishing the biotinylation process, binding of biotinylated cellulose nanowhiskers to biotinylated microtubules was studied using alexafluor 647-conjugated streptavidin. A key challenge in this study was to prevent streptavidin-induced aggregation of biotinylated cellulose. Streptavidin has four biotin binding sites and each cellulose nanowhisker in turn was functionalized with several biotin molecules. If not controlled, streptavidin will act as ‘glue’ that will bind cellulose nanowhiskers to each other. To prevent this, an excess of streptavidin was added to the biotinylated cellulose nanowhisker solution. The objective was to saturate all the biotin sites on the cellulose nanowhiskers with individual streptavidin. If every biotin molecule is attached to streptavidin (no free biotin available) then the biotinylated cellulose nanowhiskers cannot bind to each other. The unbound streptavidin was removed by centrifuging and resuspending the streptavidin-coated biotinylated cellulose nanowhiskers in BRB80 buffer. The cellulose nanowhiskers suspension was then added to 160 nM biotinylated and rhodamine-labeled microtubules and incubated for twenty minutes. The solution exhibited fluorescence under both the TRITC and Cy5 filters, indicating that rhodamine-labeled biotinylated microtubules and biotinylated cellulose bind to each other. In individual control experiments, microtubules and biotinylated microtubules coated with alexafluor 647-conjugated streptavidin were studied under the fluorescence microscope for any possible “bleed through”. Bleed through is receiving fluorescence signal of one dye through a filter used for another dye, which happens especially if very high concentrations of a dye are present or when two dyes have close emission ranges. There was no bleed through in the case of rhodamine microtubules and alexafluor 647 streptavidin (data not shown).

**Figure 4:**
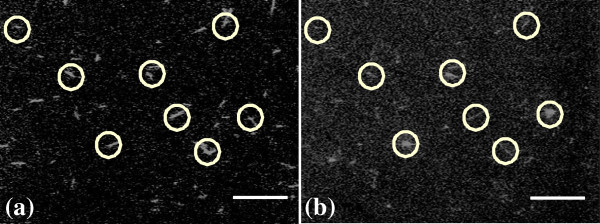
**Fluorescence studies of linked biotinylated cellulose and biotinylated microtubule via neutravidin.** (**a**) Rhodamine labeled microtubules viewed under a TRITC filter (emission centered at 630 nm) (**b**) Alexa fluor labeled cellulose viewed under a Cy5 filter (emission centered at 699 nm). Contrast was enhanced in the images above. The scale bar is 20 μm.

While it was confirmed that microtubules can be bound to cellulose, it was difficult to visualize individual microtubules binding to cellulose nanowhiskers due to the bright fluorescence signal. To visualize individual cellulose whiskers on biotinylated microtubules, biotinylated microtubules were immobilized on 3-aminopropyl triethoxysilane (APTES)-coated glass surfaces using kinesin and biotinylated cellulose nanowhiskers were then added in the presence of streptavidin (see Methods). Overlapping fluorescence was observed with TRITC and Cy5 filters, indicating the microtubules and cellulose nanowhiskers were bound to one another. This is shown in Figure 4. There was no fluorescence observed with the FITC filter. Observation of overlapping fluorescence from rhodamine-labeled biotinylated microtubules and biotinylated cellulose nanowhiskers linked to alexafluor 647-labeled streptavidin further confirmed the binding of microtubules and cellulose nanowhiskers using a biotin-avidin linkage.

**Figure 5:**
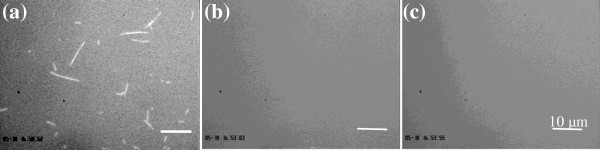
**Control experiments for linking of biotinylated cellulose with biotinylated microtubules.** (**a**) Microtubules were observed to bind to biotinylated cellulose coated surface using neutravidin. (**b**) Absence of biotinylated cellulose lead to no binding of biotinylated microtubules showing the microtubules only bound to biotinylated cellulose and not to the glass surface non-specifically. (**c**) In absence of neutravidin biotinylated microtubules did not bind to biotinylated cellulose indicating the linkage between biotinylated cellulose and biotinylated microtubule is via biotin-neutravidin. This control again verified that microtubules do not bind non-specifically to the glass surface. Contrast was enhanced in (**a**). The scale bar is 10 μm.

To further confirm the binding of biotinylated microtubules to biotinylated cellulose nanowhiskers, the above geometry was reversed (see methods). Biotinylated microtubules were then introduced into a flow cell and allowed to bind to cellulose nanowhiskers previously bound to the glass surface. The unbound microtubules were washed out with antifade solution before visualization by fluorescence microscopy. Figure 5a shows the fluorescence observed. To verify each step, particularly to confirm that cellulose nanowhiskers and microtubules bind together and that there was no background fluorescence signal or non-specific binding (between microtubules and glass), several control experiments were performed. To verify that biotinylated microtubules bound only to biotinylated cellulose nanowhiskers via biotin-neutravidin and not nonspecifically to the glass, a similar experiment was performed, except that biotinylated cellulose nanowhiskers were not introduced into the flow cell. No rhodamine fluorescence was recorded in this case, confirming that no microtubules bound to the glass surface in the absence of biotinylated cellulose nanowhiskers (Figure 5b). To verify that microtubules bind to the cellulose nanowhiskers through biotin-avidin interactions, a control experiment was carried using the same procedures but leaving out the neutravidin. Again no microtubule binding was observed either to the glass surface or to the cellulose nanowhiskers, confirming that binding was mediated by a biotin- neutravidin linkage (Figure 5c).

## Conclusions

Asters of microtubule seeds were immobilized on glass surfaces and the microtubules extended to establish percolated networks of microtubules. Biotinylated cellulose nanowhiskers were successfully linked to biotinylated microtubules via neutravidin and the specificity of this interaction confirmed by several control experiments. This work confirms the viability of using cytoskeletal filaments and motor proteins to create complex geometries and using these networks as templates for organizing nanoparticles and nanofibers such as cellulose nanowhiskers.

## Competing interests

The authors declare that they have no competing interests.

## Authors’ contribution

VV carried out all the experiments. JMC, VV, NRB and WOH contributed to the manuscript preparation. VV and JMC conceived the studies and designed the experiments. JMC, VV and WOH contributed to the microtubules polymerization, biotinylation and fluorescence studies discussions. JMC, VV and NRB contributed in cellulose hydrolysis discussion. All authors have read and approved the final manuscript.
